# Supplementary material for: Chiral terahertz wave emission from the Weyl semimetal TaAs
Source: Nat Commun. 2020 Feb 5;11:720. doi: 10.1038/s41467-020-14463-1 (PMC7002692; doi:10.1038/s41467-020-14463-1)
Supplement: Supplementary file 1 — Supplementary Information [file 41467_2020_14463_MOESM1_ESM.pdf]

Supplementary Information: Chiral terahertz wave emission from the Weyl semimetal TaAs

Gao et al.

Supplementary Figure 1

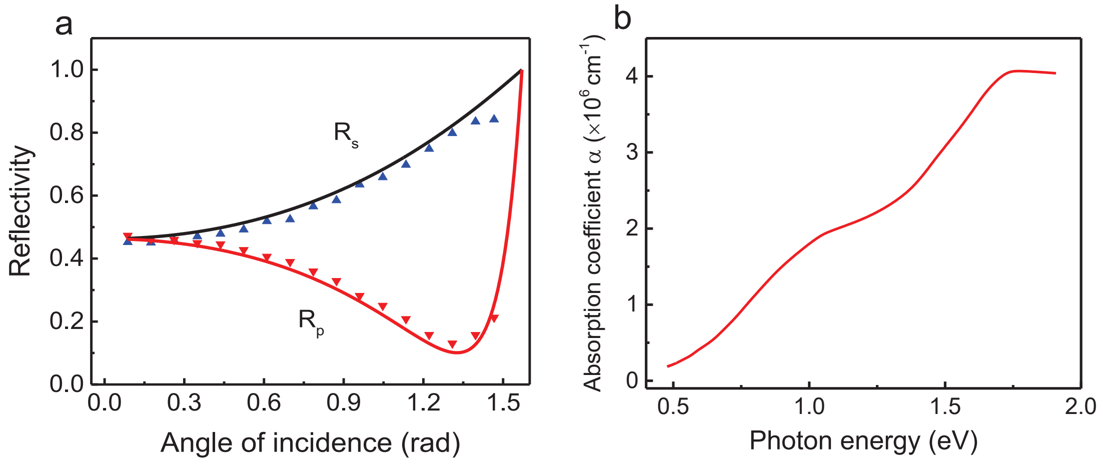

Supplementary Figure 1. **Optical properties in the high photon energy range.** **a**, Experimental reflectivities (800 nm) for *p*- and *s*-polarization as a function of the incident angle. The solid curves are the fitted values. **b**, Absorption coefficient as a function of the photon energy extracted from Ref. [2].

Supplementary Figure 2

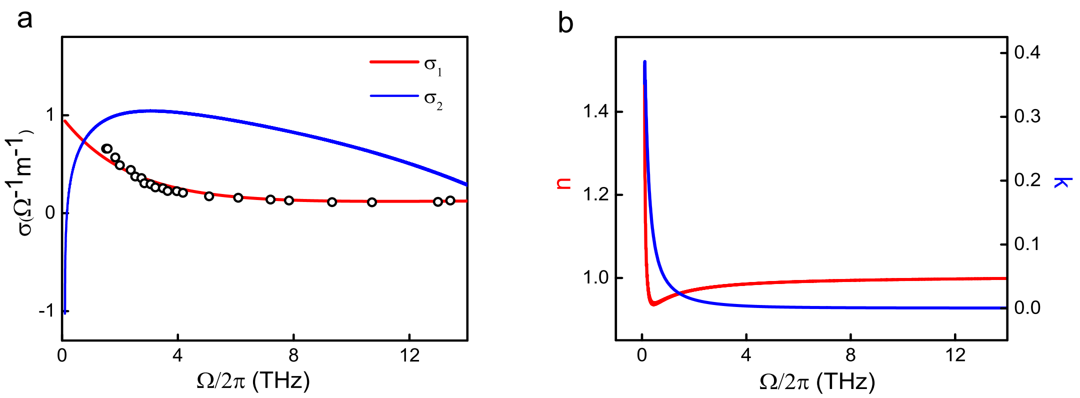

Supplementary Figure 2. Calculated conductivity ($\sigma = \sigma_1 + i\sigma_2$) and complex refractive index ($\tilde{N} = n + ik$) at the THz frequency range. The open circles are experimental data from Ref. [3].

Supplementary Figure 3

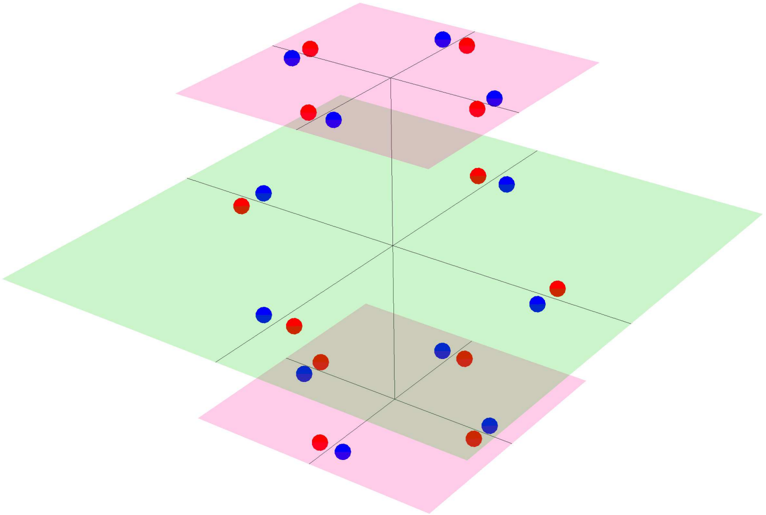

Supplementary Figure 3. **Illustration of the 24 Weyl cones of TaAs in momentum space.** The colors represent chirality.

Supplementary Figure 4

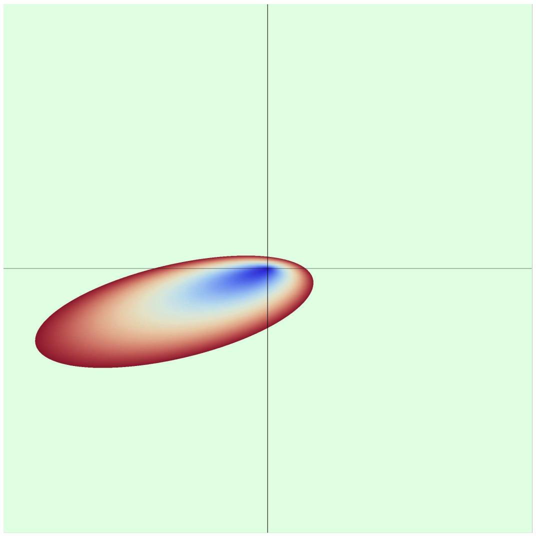

Supplementary Figure 4. Sketch of a tilted and anisotropic Weyl cone. The shading represents energy. The axes are the principal axes of the untilted Hamiltonian; the origin is the Weyl point. The tilt parameter is $\alpha = 5/6$.

Supplementary Figure 5

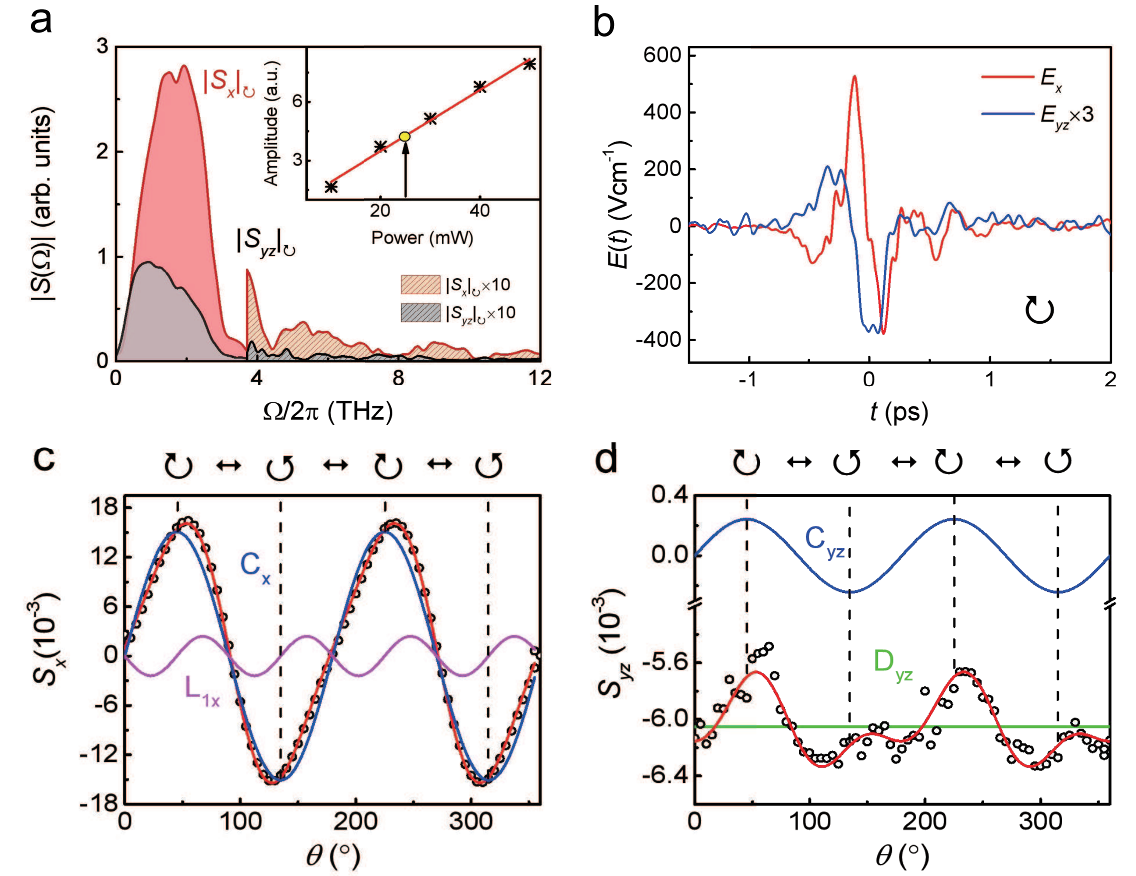

Supplementary Figure 5. **Far (or near) field THz signals in the frequency (or time) domain and their polarization dependence.** **a**, Fourier transform spectra of $S_{\mathrm{x}}(t)$ and $S_{\mathrm{yz}}(t)$ measured at 25 mW in the main text. **b**, The corresponding THz near fields $\mathbf{E}_{\mathrm{x}}(t)$ and $\mathbf{E}_{\mathrm{yz}}(t)$. The pump light is right-handed circularly polarized. Inset shows the THz signal is linearly dependent on the pump power. **c** and **d** display the EO signals $S_{\mathrm{x}}(t=-0.12\text{ ps})$ and $S_{\mathrm{yz}}(t=-0.08\text{ ps})$ (near the peak values) as a function of the rotation angle of the quarter-wave plate, θ. The red lines are fitted results obtained using Eq.(1) in the main text. The blue and pink lines show the two dominant terms.

Supplementary Figure 6

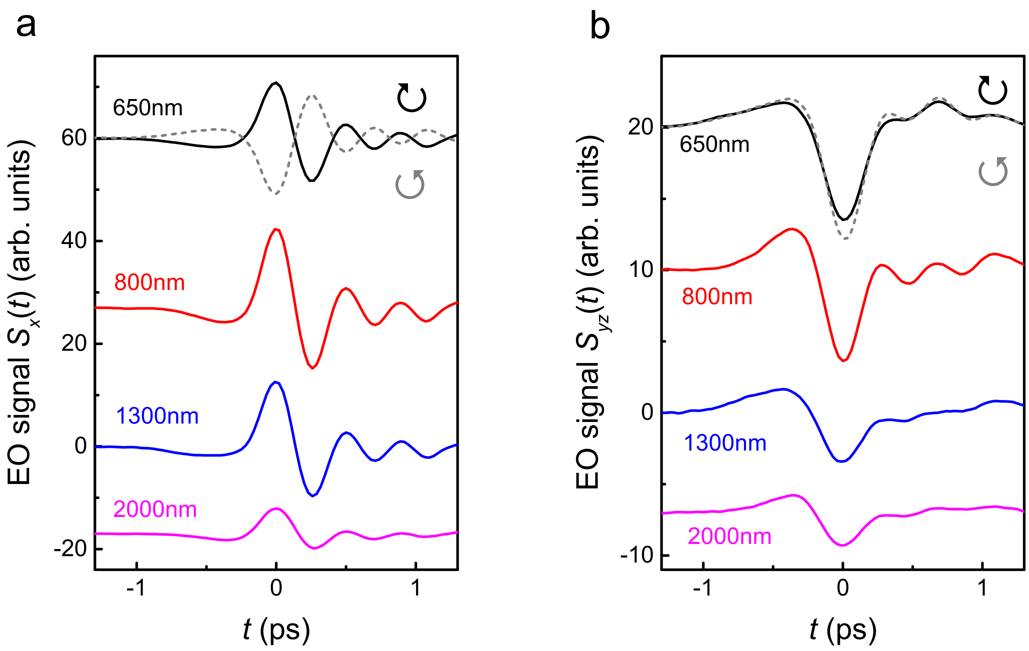

Supplementary Figure 6. The electro-optic (EO) signals at different excitation wavelengths. THz EO signals $S_\mathrm{x}(t)$ and $S_\mathrm{yz}(t)$ under excitation of circularly polarized light for (112) face. Solid and dashed lines correspond to different circular polarization.

Supplementary Figure 7

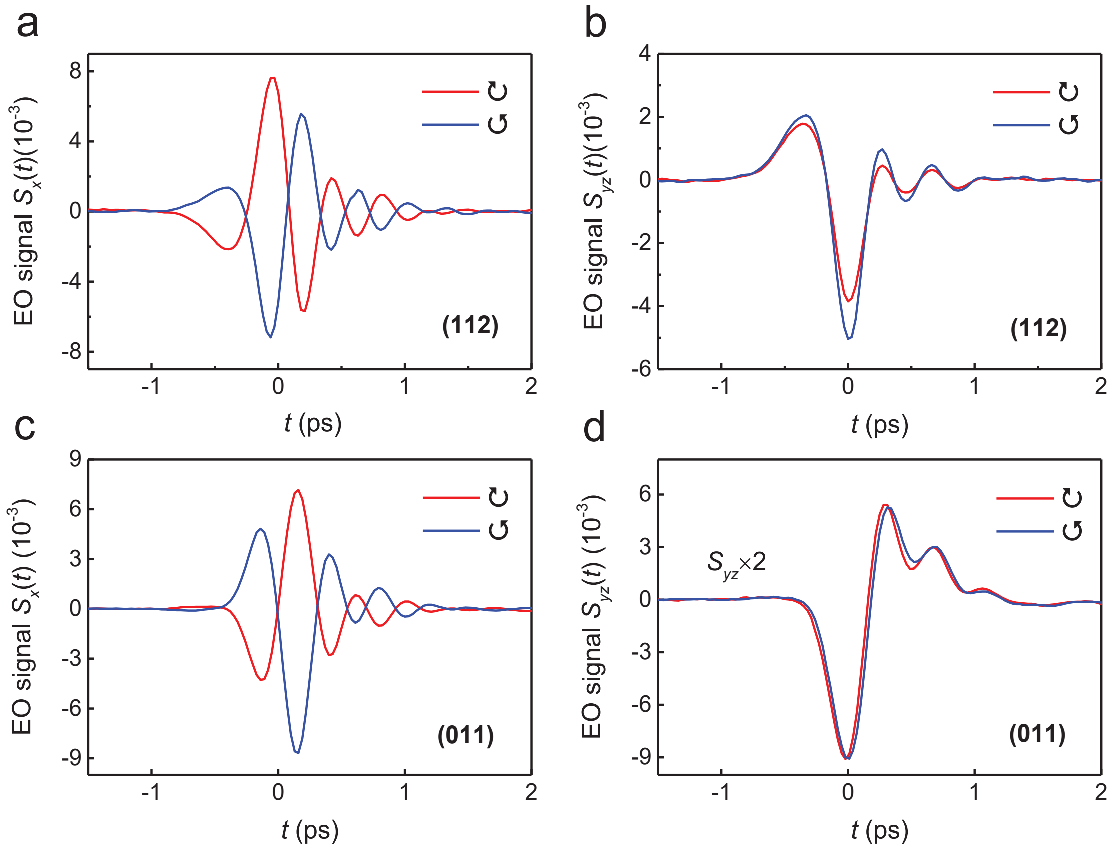

Supplementary Figure 7. **THz electro-optic signals at $\Theta \simeq 45°$.** $S_x(t)$ and $S_{yz}(t)$ for (112) and (011) faces.

Supplementary Figure 8

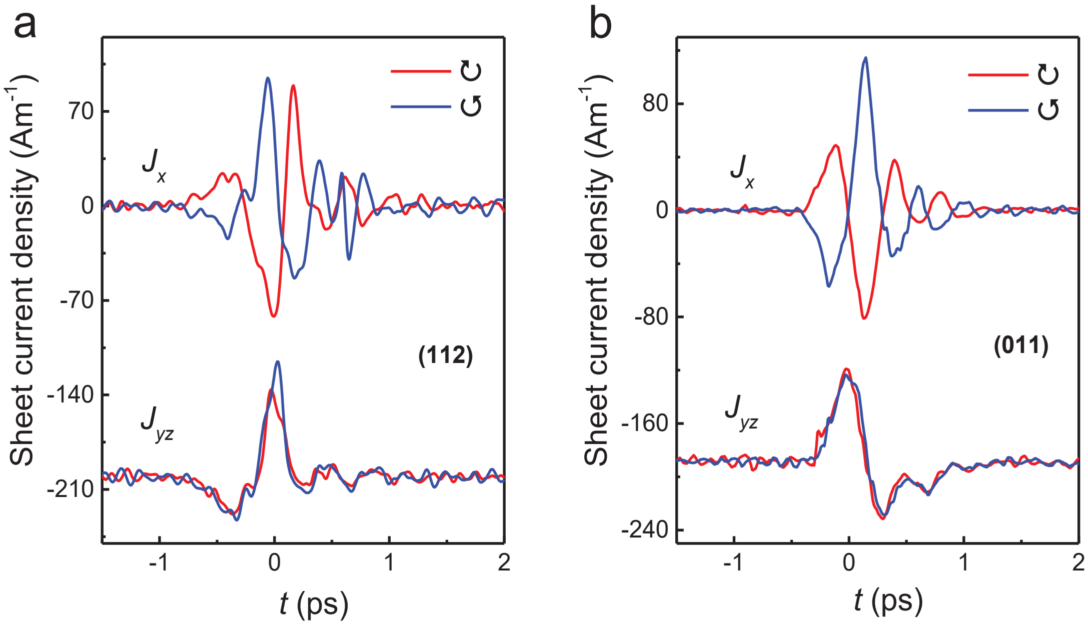

Supplementary Figure 8. The corresponding ultrafast sheet current densities at $\Theta \simeq 45°$. $J_{\mathrm{x}}(t)$ and $J_{\mathrm{yz}}(t)$ for (112) and (011) faces.

Supplementary Figure 9

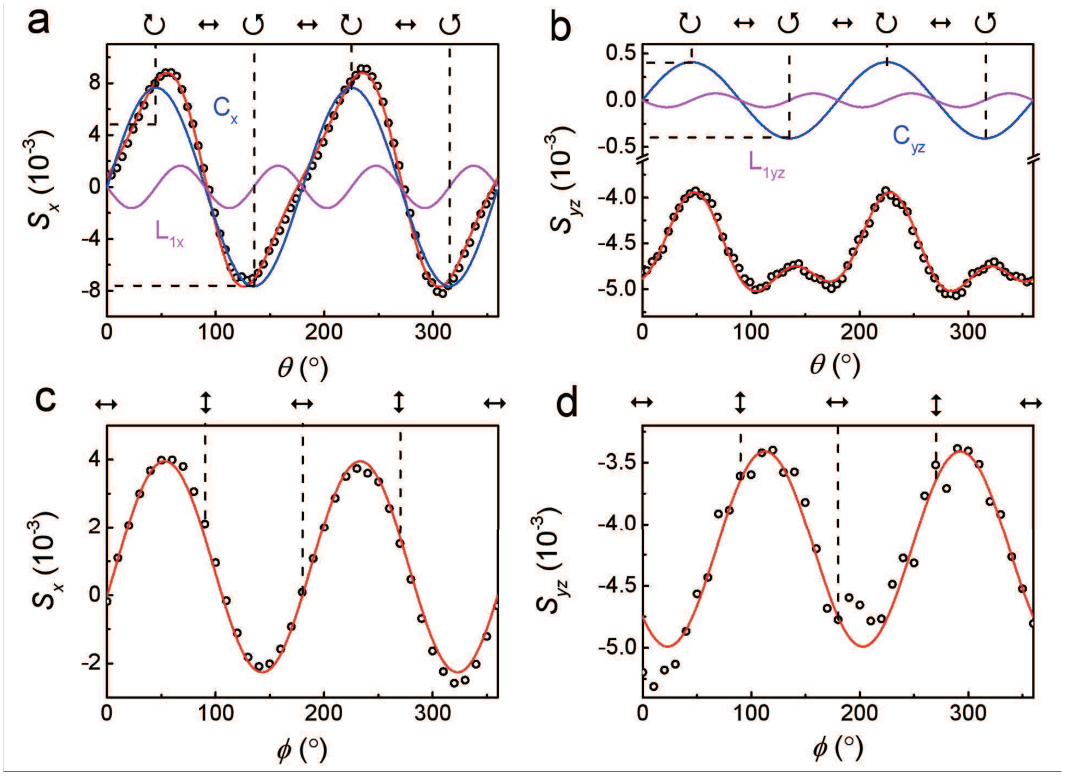

Supplementary Figure 9. **Polarization dependence of the electro-optic signals for (112) face with $\Theta \simeq 45°$. a** and **b**, Peak amplitudes of the electro-optic signals S_x and S_{yz} as a function of the rotation angle of quater-wave plate, θ. The colored solid lines are fitting results using Eq.(1) of the main text. **c** and **d**, The peak amplitudes of electro-optic signals S_x and S_{yz} as a function of the linear-polarization angle, ϕ. Red solid lines are fitting results using Eq.(10) of the main text.

Supplementary Figure 10

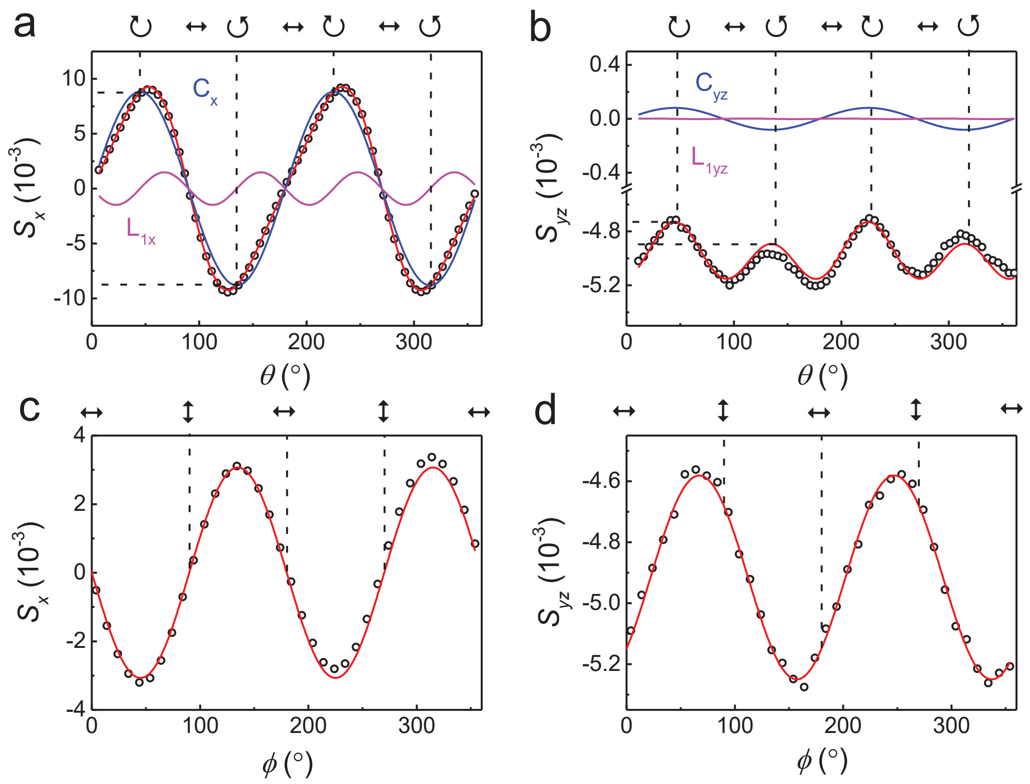

Supplementary Figure 10. **Polarization dependence of the electro-optic signals for (011) face with $\Theta \simeq 45°$.** **a** and **b**, Peak amplitudes of the electro-optic signals S_x and S_yz as a function of the rotation angle of quater-wave plate, θ. The colored solid lines are fitting results using Eq.(1) of the main text. **c** and **d**, The peak amplitude of electro-optic signals S_x and S_yz as a function of the linear-polarization angle, ϕ. Red solid lines are fitting results using Eq.(10) of the main text. All data for (011) face with $\Theta \simeq 45°$.

Supplementary Figure 11

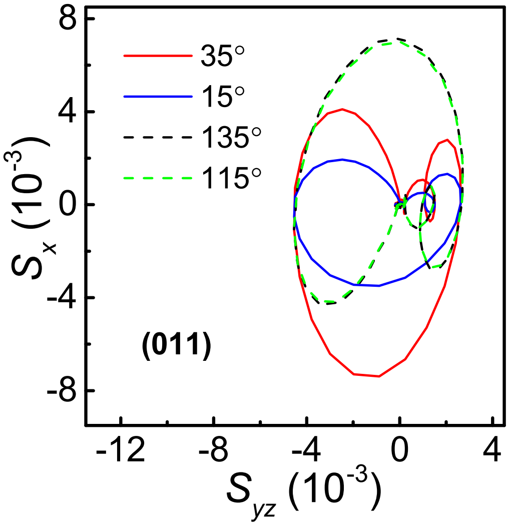

Supplementary Figure 11. **The polarization trajectory ($S_\mathbf{x}(t)$,$S_\mathbf{yz}(t)$) for different elliptically polarized pump light with various θ for $\Theta = 45°$.** The solid and dashed lines represent different chiralities. The THz pulse is close to circularly polarized at $\theta = 15°$.

Supplementary Figure 12

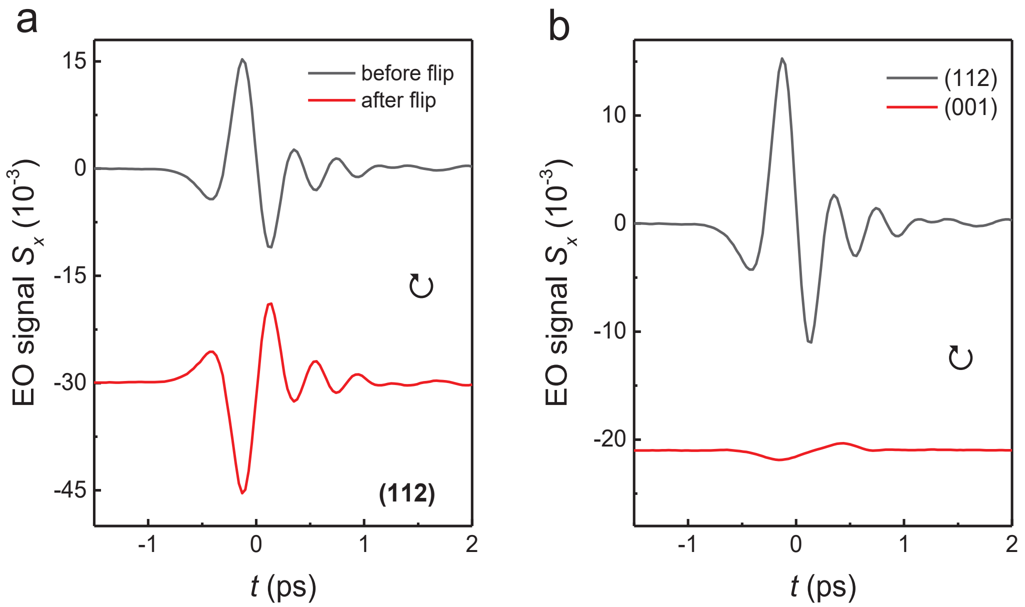

Supplementary Figure 12. The electro-optic (EO) signals for different sample directions. a, Comparison of the THz signals $S_{\mathrm{x}}(t)$ before and after flipping the c-axis direction by rotating the sample with an angle of 180 degree for (112) face. **b**, Comparison of the THz signal strength $S_{\mathrm{x}}(t)$ between (112) and (001) face.

Supplementary Figure 13

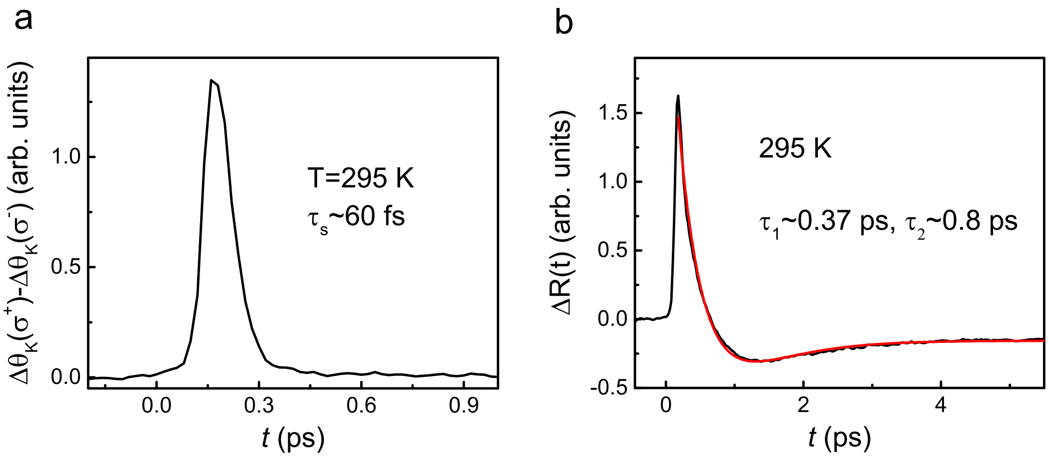

Supplementary Figure 13. **Transient reflectivity and Kerr rotation signals for TaAs.** **a**, Transient reflectivity $\Delta R(t)$ for TaAs. Red line is the fitted curve using two exponential decay function. τ_1, with a time of ∼370 fs, is consistent with previous work[4]. **b**, Time-resolved Kerr rotation $\Delta\theta_{\rm K}(t)$ upon excitation of circularly polarized light for TaAs. Based on the experimental data, the spin relaxation time $\tau_{\rm s}$ is ∼60 fs.

Supplementary Figure 14

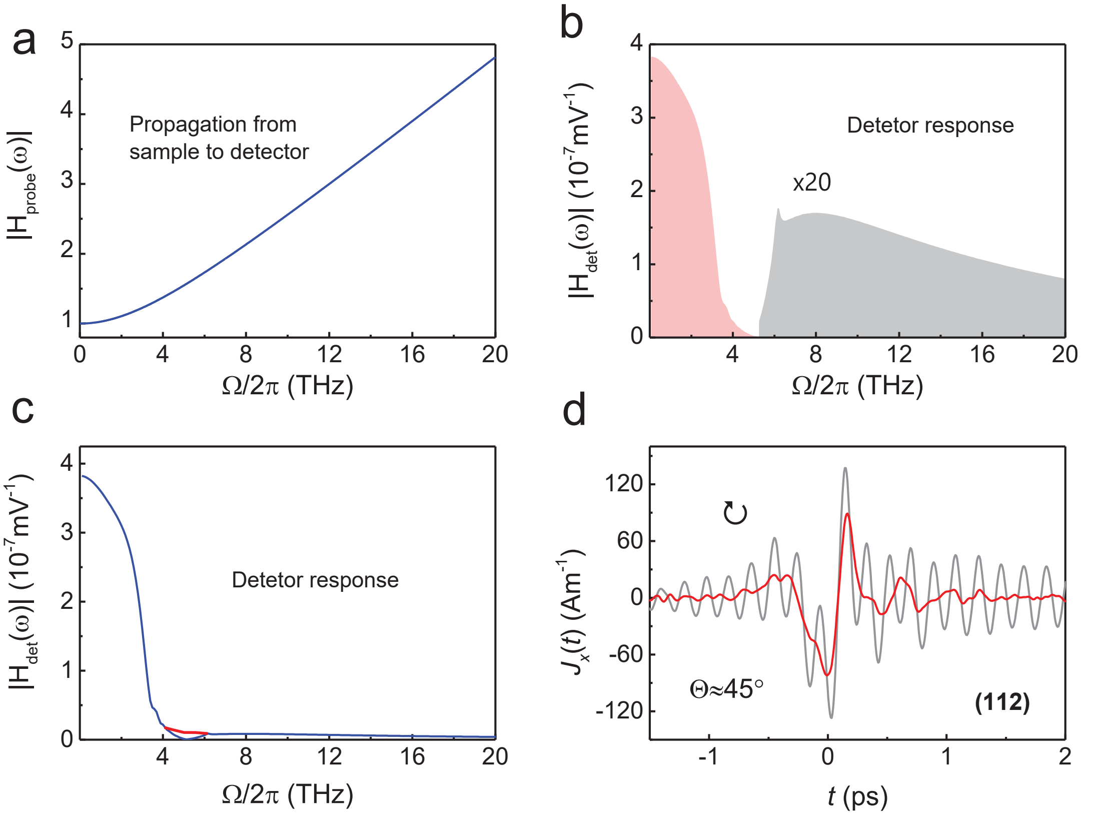

Supplementary Figure 14. **The calculated transfer functions.** **a**, Transfer function $h_{\text{prop}}(\Omega)$ describing the propagation from the sample to the electro-optic detection crystal [5]. The absorption dip around ∼5 THz arises from the Restrahlen band of the ZnTe detection crystal. **b**, Transfer function $h_{\text{det}}(\Omega)$ of the electro-optic detection consisting of a 400 μm thick ZnTe(110) crystal in conjunction with a 80 fs, 800 nm sampling pulse [6]. **c**, In order to increase the signal-to-noise ratio worsened by the dip around ∼5 THz, we used a set of smoothed data to replace the original $h_{\text{det}}(\Omega)$ between 4.2 and 6.2 THz, indicated by the red line. **d**, Calculated ultrafast photocurrents using the original $h_{\text{det}}(\Omega)$ (gray line) and the modified $h_{\text{det}}(\Omega)$ (red line), respectively. The latter clearly gives an enhancement of signal-to-noise ratio but does not lose the overall signal profile.

Supplementary Figure 15

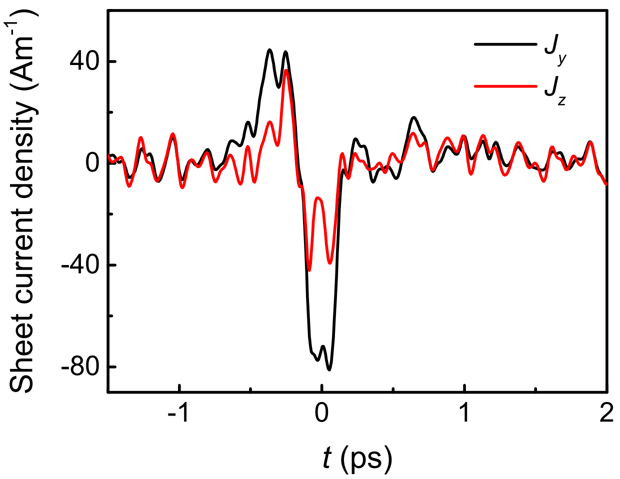

Supplementary Figure 15. **Calculated sheet current densities $J_{\mathbf{y}}(t)$ and $J_{\mathbf{z}}(t)$.**

Supplementary Note 1

Symmetry analysis. The third-rank tensor $\xi_{\lambda\mu\nu}$ is nonzero only in systems with broken inversion symmetry, which can apply for the bulk TaAs. According to the $4mm$ crystal symmetry, there are only three independent tensor elements:$\sigma_1 = \xi_{\rm zzz}$, $\sigma_2 = \xi_{\rm zxx} = \xi_{\rm zyy}$ and $\sigma_3 = \xi_{\rm xzx} = \xi_{\rm yzy} = \xi_{\rm xxz} = \xi_{\rm yyz}$. The other tensor elements are zero [1]. We also define $I_0 = Ff^*$. The refractive angle β is obtained by the Snell's law: $\sin\beta = \sin\Theta/n_{\rm p}$, where the angle of incidence Θ and the refractive index of pump light $n_{\rm p} \simeq 3.16$.

According to the equation in the 'Methods' section, we are able to solve the photocurrent components due to the LPGE as a function of the linear polarization angle ϕ for different faces. During the calculations, without losing the generality we omitted the frequency integral, the Fresnel coefficients in front of each equation and the prefactor 2 before the integral.

(1) For (011) face

$$\begin{aligned} j_{\rm x} &= \sigma_3 \sin(\beta + \pi/4)\sin(2\phi) I_0 \\ j_{\rm y} &= \frac{1}{2\sqrt{2}}(A_1\cos^2\phi + B_1\sin^2\phi) I_0 \\ j_{\rm z} &= \frac{1}{2\sqrt{2}}(A_2\cos^2\phi + B_2\sin^2\phi) I_0 \end{aligned} \tag{1}$$

where

$$A_1 = 2\sigma_3 \cos(2\beta) + \sigma_2[1 - \sin(2\beta)] + \sigma_1[\sin(2\beta) + 1]$$

$$B_1 = 2\sigma_2$$

$$A_2 = \sigma_3(2\sin^2\beta - 2\cos^2\beta) + \sigma_2[\sin^2\beta - \sin(2\beta) + \cos^2\beta] + \sigma_1[\sin^2\beta + \sin(2\beta) + \cos^2\beta]$$

$$B_2 = 2\sigma_2$$

(2)

(2) For (112) face

$$j_{\mathrm{x}} = \frac{1}{12\sqrt{6}}[A_1\cos^2\phi + B_1\sin^2\phi + C_1\sin(2\phi)]I_0$$
$$j_{\mathrm{y}} = \frac{1}{6\sqrt{6}}[A_2\cos^2\phi + B_2\sin^2\phi + C_2\sin(2\phi)]I_0$$
$$j_{\mathrm{z}} = \frac{1}{3\sqrt{6}}[A_3\cos^2\phi + B_3\sin^2\phi + C_3\sin(2\phi)]I_0$$

(3)

where

$$A_1 = a_1^{(1)}\sigma_3 + a_2^{(1)}\sigma_2 + a_3^{(1)}\sigma_1$$

$$B_1 = 2(10\sigma_3 + 5\sigma_2 + \sigma_1)$$

$$C_1 = c_1^{(1)}\sigma_3 + c_2^{(1)}\sigma_2 + c_3^{(1)}\sigma_1$$

$$a_1^{(1)} = -4 - 16\sin\beta\cos\beta - 12\sin^2\beta$$

$$a_2^{(1)} = 4 - 8\sin\beta\cos\beta + 6\cos^2\beta$$

$$a_3^{(1)} = 2 + 8\sin\beta\cos\beta + 6\cos^2\beta$$

$$c_1^{(1)} = 8\cos\beta + 16\sin\beta$$

$$c_2^{(1)} = -2\cos\beta - 4\sin\beta$$

$$c_3^{(1)} = 2\cos\beta + 4\sin\beta$$

(4)

$$
\begin{aligned}
A_2 &= a_1^{(2)}\sigma_3 + a_2^{(2)}\sigma_2 + a_3^{(2)}\sigma_1 \\
B_2 &= (-2\sigma_3 + 5\sigma_2 + \sigma_1) \\
C_2 &= c_1^{(2)}\sigma_3 + c_2^{(2)}\sigma_2 + c_3^{(2)}\sigma_1 \\
a_1^{(2)} &= 10\text{cos}^2\beta + 16\text{sin}\beta\text{cos}\beta - 8\text{sin}^2\beta \\
a_2^{(2)} &= 3\text{cos}^2\beta - 4\text{sin}\beta\text{cos}\beta + 2 \\
a_3^{(2)} &= 1 + 4\text{sin}\beta\text{cos}\beta + 3\text{sin}^2\beta \\
c_1^{(2)} &= 4\text{cos}\beta - 4\text{sin}\beta \\
c_2^{(2)} &= -1\text{cos}\beta - 2\text{sin}\beta \\
c_3^{(2)} &= 1\text{cos}\beta + 2\text{sin}\beta
\end{aligned}
\tag{5}
$$

$$
\begin{aligned}
A_3 &= a_1^{(3)}\sigma_3 + a_2^{(3)}\sigma_2 + a_3^{(3)}\sigma_1 \\
B_3 &= (-2\sigma_3 + 5\sigma_2 + \sigma_1) \\
C_3 &= \frac{1}{2}(c_1^{(3)}\sigma_3 + c_2^{(3)}\sigma_2 + c_3^{(3)}\sigma_1) \\
a_1^{(3)} &= -2\text{cos}^2\beta - 2\text{sin}\beta\text{cos}\beta + 4\text{sin}^2\beta \\
a_2^{(3)} &= 3\text{cos}^2\beta - 4\text{sin}\beta\text{cos}\beta + 2 \\
a_3^{(3)} &= 1 + 4\text{sin}\beta\text{cos}\beta + 3\text{sin}^2\beta \\
c_1^{(3)} &= -4\text{cos}\beta - 2\text{sin}\beta \\
c_2^{(3)} &= -2\text{cos}\beta - 4\text{sin}\beta \\
c_3^{(3)} &= 2\text{cos}\beta + 4\text{sin}\beta
\end{aligned}
\tag{6}
$$

Supplementary Note 2

Penetration depth of the pump light. We performed a static reflectivity measurement to determine the pump light (800 nm) penetration depth. We measured the reflectivities for p- and s-polarizations ($R_{\rm P}$, $R_{\rm S}$) as a function of the incident angle. The experimental data and the fitting results using the complex Fresnel equations for lossy materials are shown in Supplementary Fig. 1(a). Notice that a significant Brewster-like dip is observed at roughly 80 degrees, which is well captured by the fitting. From the fitting we extract the real and imaginary parts of the refractive index ($\tilde{N}_{\rm p} = n_{\rm p} + ik_{\rm p}$): $n_{\rm p} \simeq 3.16$, $k_{\rm p} \simeq 2.5$. The latter gives a penetration depth of $\sim$25 nm, which is consistent with the dielectric constants calculated in Ref.[2] (see Supplementary Fig. 1(b)). This value also means that generation of the ultrafast photocurrents only happen inside a region very close to the top surface.

Supplementary Note 3

Refractive index in THz frequency range. We obtained the refractive index in THz frequency range by calculating the dielectric constant of TaAs from the experimental conductivity $\sigma_1(\Omega)$ [3]. During the calculations, the Kramers-Kronig relationship was employed. Supplementary Fig. 2 shows the calculated results.

Supplementary Note 4

More details for calculations of the helicity-dependent photocurrent. Based on the assumption that once the photons enter the material they will induce excitations with unit probability, for a single Weyl cone, we

can write

$$\sum_{\mathrm{i}} |\langle s_{\mathrm{i}}|\hat{H}_{\mathrm{EM}}|q_{-}\rangle|^2 = \mathrm{Tr}(\hat{H}_{\mathrm{EM}}\,|q_{-}\rangle\,\langle q_{-}|\,\hat{H}^{*}_{\mathrm{EM}}). \tag{7}$$

If we define the dispersion relation

$$\sum a^{\mu\nu}\, p_{\mu} p_{\nu} = 0 \tag{8}$$

and compare it to Eq.(2), we find that

$$a^{00} = 1 \tag{9}$$

$$a^{0\mathrm{i}} = -2v_{\mathrm{t}}^{\mathrm{i}} \tag{10}$$

$$a^{\mathrm{ij}} = v_{\mathrm{t}}^{\mathrm{i}}\, v_{\mathrm{t}}^{\mathrm{j}} - v_{\mathrm{a}}^{\mathrm{i}}\, v_{\mathrm{a}}^{\mathrm{j}} \tag{11}$$

To calculate Eq. (7), we first find

$$|q_{-}\rangle\,\langle q_{-}| = \frac{\sigma_0\sqrt{-\det(\hat{H}_{\mathrm{W}})} - \hat{H}_{\mathrm{W}}}{2\sqrt{-\det(\hat{H}_{\mathrm{W}})}} \tag{12}$$

$$-\det(\hat{H}_{\mathrm{W}}) = (v_{\mathrm{t}}^{\mathrm{i}}\, v_{\mathrm{t}}^{\mathrm{j}} - a^{\mathrm{ij}})q_{\mathrm{i}}q_{\mathrm{j}} \tag{13}$$

$$\mathrm{Tr}(\hat{H}_{\mathrm{EM}}\,|q_{-}\rangle\,\langle q_{-}|\,\hat{H}^{*}_{\mathrm{EM}}) = (v_{\mathrm{t}}^{\mathrm{i}}\, v_{\mathrm{t}}^{\mathrm{j}} - a^{\mathrm{ij}})\varepsilon_{\mathrm{i}}\varepsilon_{\mathrm{j}}^{*} + v_{\mathrm{t}}^{\mathrm{i}}\, v_{\mathrm{t}}^{\mathrm{j}}\varepsilon_{\mathrm{i}}\varepsilon_{\mathrm{j}}^{*} - \frac{\mathrm{Tr}(\varepsilon_{\mathrm{i}}\, v_{\mathrm{a}}^{\mathrm{i}}\,\sigma_{\mathrm{a}}\, v_{\mathrm{b}}^{\mathrm{j}}\, q_{\mathrm{j}}\,\sigma_{\mathrm{b}}\,\varepsilon_{\mathrm{k}}^{*}\, v_{\mathrm{c}}^{\mathrm{k}}\,\sigma_{\mathrm{c}})}{2\sqrt{(v_{\mathrm{t}}^{\mathrm{i}}\, v_{\mathrm{t}}^{\mathrm{j}} - a^{\mathrm{ij}})q_{\mathrm{i}}q_{\mathrm{j}}}} - 2\frac{(v_{\mathrm{t}}^{\mathrm{i}}\, v_{\mathrm{t}}^{\mathrm{k}} - a^{\mathrm{ik}})\varepsilon_{\mathrm{i}}q_{\mathrm{k}}v_{\mathrm{t}}^{\mathrm{j}}\varepsilon_{\mathrm{j}}}{\sqrt{(v_{\mathrm{t}}^{\mathrm{i}}\, v_{\mathrm{t}}^{\mathrm{j}} - a^{\mathrm{ij}})q_{\mathrm{i}}q_{\mathrm{j}}}} \tag{14}$$

where ε_{i} is the polarization of the photon.

$$\begin{aligned}\mathrm{Tr}(\varepsilon_{\mathrm{i}}\, v_{\mathrm{a}}^{\mathrm{i}}\,\sigma_{\mathrm{a}}\, v_{\mathrm{b}}^{\mathrm{j}}\, q_{\mathrm{j}}\,\sigma_{\mathrm{b}}\,\varepsilon_{\mathrm{k}}^{*}\, v_{\mathrm{c}}^{\mathrm{k}}\,\sigma_{\mathrm{c}}) &= 2\mathrm{i}\epsilon_{\mathrm{abc}}\,\varepsilon_{\mathrm{i}}\, v_{\mathrm{a}}^{\mathrm{i}}\, v_{\mathrm{b}}^{\mathrm{j}}\, q_{\mathrm{j}}\,\varepsilon_{\mathrm{k}}^{*}\, v_{\mathrm{c}}^{\mathrm{k}} & (15)\\ &= 2\mathrm{i}\det\left[(\varepsilon q \varepsilon^{*})(v_{\mathrm{a}}^{\mathrm{i}})\right] & (16)\\ &= 2\mathbf{q}.\mathbf{L}\det(v_{\mathrm{a}}^{\mathrm{i}}). & (17)\end{aligned}$$

Now, since $v^{\mathrm{i}}_{\mathrm{a}} v^{\mathrm{i}}_{\mathrm{b}} = v^{\mathrm{i}}_{\mathrm{t}}\, v^{\mathrm{j}}_{\mathrm{t}} - a^{\mathrm{ij}}$, $\det(v^{\mathrm{i}}_{\mathrm{a}}) = \chi\sqrt{\det(v^{\mathrm{i}}_{\mathrm{t}}\, v^{\mathrm{j}}_{\mathrm{t}} - a^{\mathrm{ij}})}$, where χ is the chirality of the Weyl fermion. Eq. (7) becomes

$$\sum_{\mathrm{i}} |\langle s_{\mathrm{i}}|\hat{H}_{\mathrm{EM}}|q\rangle|^2 = (2v^{\mathrm{i}}_{\mathrm{t}}\, v^{\mathrm{j}}_{\mathrm{t}} - a^{\mathrm{ij}})\varepsilon_{\mathrm{i}}\varepsilon^{*}_{\mathrm{j}} + \frac{\chi \mathbf{q}.\mathbf{L}\sqrt{\det(v^{\mathrm{i}}_{\mathrm{t}}\, v^{\mathrm{j}}_{\mathrm{t}} - a^{\mathrm{ij}})}}{\sqrt{(v^{\mathrm{i}}_{\mathrm{t}}\, v^{\mathrm{j}}_{\mathrm{t}} - a^{\mathrm{ij}})q_{\mathrm{i}}q_{\mathrm{j}}}} - 2\frac{(v^{\mathrm{i}}_{\mathrm{t}}\, v^{\mathrm{k}}_{\mathrm{t}} - a^{\mathrm{ik}})\varepsilon_{\mathrm{i}} q_{\mathrm{k}} v^{\mathrm{j}}_{\mathrm{t}}\varepsilon_{\mathrm{j}}}{\sqrt{(v^{\mathrm{i}}_{\mathrm{t}}\, v^{\mathrm{j}}_{\mathrm{t}} - a^{\mathrm{ij}})q_{\mathrm{i}}q_{\mathrm{j}}}} \tag{18}$$

TaAs possesses invariance with respect to time reversal t. Time reversal takes momentum $\mathbf{k}$ to $-\mathbf{k}$ and σ_{a} to $-\sigma_{\mathrm{a}}$, so the chirality is preserved under t-reversal. The velocity $\mathbf{v}$ that enters equation (4) is odd under time reversal. In Eq. (18), the second term is parity odd and the other two terms are parity even. When Eq. (18) is integrated and summed over the Weyl cones, only the first and third term survive. Eliminating the terms that cancel, Eq.(4) becomes

$$\begin{aligned}\mathbf{J} &= \int \mathbf{j}\, \mathrm{d}z \\ &= \frac{eI}{\hbar\omega} \frac{\sum \tau a \int \mathrm{d}^3 q\, \delta(E_{-}(q) - E_1 + \hbar\omega)\, \mathbf{v}_{-}(q)\, \frac{\chi \mathbf{q}.\mathbf{L}\sqrt{\det((E^2_{\mathrm{W}})^{\mathrm{ij}})}}{\sqrt{(E^2_{\mathrm{W}})^{\mathrm{ij}} q_{\mathrm{i}} q_{\mathrm{j}}}}}{\sum a \int \mathrm{d}^3 q\, \delta(E_{-}(q) - E_1 + \hbar\omega) \left(\left(v^{\mathrm{i}}_{\mathrm{t}} v^{\mathrm{j}}_{\mathrm{t}} + (E^2_{\mathrm{W}})^{\mathrm{ij}} \right) \varepsilon_{\mathrm{i}}\varepsilon^{*}_{\mathrm{j}} - 2\frac{(E^2_{\mathrm{W}})^{\mathrm{ik}}\varepsilon_{\mathrm{i}} q_{\mathrm{k}} v^{\mathrm{j}}_{\mathrm{t}}\varepsilon_{\mathrm{j}}}{\sqrt{(E^2_{\mathrm{W}})^{\mathrm{ij}} q_{\mathrm{i}} q_{\mathrm{j}}}} \right)}\end{aligned} \tag{19}$$

where $(E^2_{\mathrm{W}})^{\mathrm{ij}} = v^{\mathrm{i}}_{\mathrm{t}} v^{\mathrm{j}}_{\mathrm{t}} - a^{\mathrm{ij}} = v^{\mathrm{i}}_{\mathrm{a}} v^{\mathrm{j}}_{\mathrm{a}}$. The sum is over the 24 Weyl cones, as shown in Supplementary Fig. **3**.

When we integrate the numerator, we get the tensor:

$$N^{\mathrm{i}}_{(\mathrm{l})\mathrm{j}} = (E_{\mathrm{l}0} - E_{\mathrm{l}1} + \hbar\omega)^2 \left[\frac{-\tanh^{-1}\alpha + \frac{\alpha}{1-\alpha^2}}{\alpha^3} \delta^{\mathrm{i}}_{\mathrm{j}} + \frac{3\tanh^{-1}\alpha - 2\alpha - \frac{\alpha}{1-\alpha^2}}{\alpha^5} (E^2_{\mathrm{W}})^{-1}_{\mathrm{jk}} v^{\mathrm{k}}_{\mathrm{t}} v^{\mathrm{i}}_{\mathrm{t}} \right] \tag{20}$$

and integrating the denominator, we get the tensor

$$D^{\mathrm{ij}}_{(\mathrm{l})} = (E_{\mathrm{l0}} - E_{\mathrm{l1}} + \hbar\omega)^2 \frac{2}{1-\alpha^2} \frac{(E^2_{\mathrm{W}})^{\mathrm{ij}} - v^{\mathrm{i}}_{\mathrm{t}} v^{\mathrm{j}}_{\mathrm{t}}}{\sqrt{\det(E^2_{\mathrm{W}})^{\mathrm{ij}}}} \tag{21}$$

where the parameter α, which quantifies the "tiltedness" of the cone, is defined as:

$$\alpha^2 = (E^2_{\mathrm{W}})^{-1}_{\mathrm{ij}} v^{\mathrm{i}}_{\mathrm{t}} v^{\mathrm{j}}_{\mathrm{t}} \tag{22}$$

Substituting these tensors into Eq. (19), we get

$$J_{\mathrm{i}}(\omega, \mathbf{k}_{\mathrm{p}}, \varepsilon) = \frac{-eI}{\hbar\omega} \frac{\sum_{\mathrm{l}} \tau_{\mathrm{l}} a_{\mathrm{l}} \chi_{\mathrm{l}} N^{\mathrm{i}}_{(\mathrm{l})\mathrm{j}} L^{\mathrm{j}}}{\sum_{\mathrm{l}} a_{\mathrm{l}} D^{\mathrm{ij}}_{(\mathrm{l})} \varepsilon_{\mathrm{i}} \varepsilon^*_{\mathrm{j}}}, \tag{23}$$

Because of the tetragonal symmetry of TaAs, the only surviving component of the tensor $\sum_{\mathrm{l}} \tau_{\mathrm{l}} a_{\mathrm{l}} \chi_{\mathrm{l}} N^{\mathrm{i}}_{(\mathrm{l})\mathrm{j}}$ would come from the antisymmetric $x - y$ component $N^{\mathrm{x}}_{(\mathrm{l})\mathrm{y}} - N^{\mathrm{y}}_{(\mathrm{l})\mathrm{x}}$. The diagonal components cancel for left and right handed cones, and all other off-diagonal components cancel due to the 4-fold rotational symmetry.

The only contribution to the antisymmetric part of $N^{\mathrm{i}}_{(\mathrm{l})\mathrm{j}}$ is from the second term $(E^2_{\mathrm{W}})^{-1}_{\mathrm{jk}} v^{\mathrm{k}}_{\mathrm{t}} v^{\mathrm{i}}_{\mathrm{t}}$; it is non-zero only if $v^{\mathrm{i}}_{\mathrm{t}}$ is not along any of the principal axes of $(E^2_{\mathrm{W}})^{\mathrm{ij}} = v^{\mathrm{i}}_{\mathrm{a}} v^{\mathrm{j}}_{\mathrm{a}}$, i.e. the tilt of the Hamiltonian is not along any principal axis of the untilted part, as shown in Supplementary Fig. 4.

Supplementary Note 5

Extra data. Supplementary Fig. 5 shows the Fourier transform of $S_{\mathrm{x}}(t)$ and $S_{\mathrm{yz}}(t)$, THz near fields $\mathbf{E}_{\mathrm{x}}(t)$ and $\mathbf{E}_{\mathrm{yz}}(t)$, and time-dependent fitting parameters in Eq. (1) of the main text. At pump power of 50 mW, the dynamic range of $\mathbf{S}(t)$ and the peak electric field of the THz pulse nearly reach 60 dB and 1 kVcm^{-1}, respectively. In terms of the THz emission

efficiency, TaAs(112) THz emitter roughly has a value smaller by a factor of ∼5 than that of the ZnTe(110) THz emitter with a thickness of 1 mm.

Supplementary Fig. 6 shows THz EO signals $S_{\mathrm{x}}(t)$ and $S_{\mathrm{yz}}(t)$ under excitation of circularly polarized light at several typical wavelengths for (112) face. In contrast to the $S_{\mathrm{yz}}(t)$ component nearly independent on the incident polarization state, the $S_{\mathrm{x}}(t)$ components taken with right- (↻) and left-handed (↺) circularly polarized light are completely out of phase for all the wavelengths investigated. Note that only one typical example (650 nm) is shown inside the figure.

Supplementary Fig. 7 and Supplementary Fig. 8 show the THz signals and their corresponding ultrafast photocurrents for (112) and (011) faces at $\Theta \simeq 45°$. For (011) face, $\hat{\mathbf{x}}$ is along [100] direction.

Supplementary Fig. 9 and Supplementary Fig. 10 show the results of peak THz signals depending on the $\lambda/4$ and $\lambda/2$ waveplates rotation for (112) and (011) faces at $\Theta = 45°$, respectively.

Supplementary Fig. 11 illustrates the polarization trajectory ($S_{\mathrm{x}}(t)$,$S_{\mathrm{yz}}(t)$) for different polarized pump light with various θ. The THz pulse is close to circularly polarized at $\theta = 15°$.

Supplementary Fig. 12 illustrates the electro-optic signals $S_{\mathrm{x}}(t)$ before and after the direction reversal of c-axis, and the comparison of THz signals between (112) and (001) faces.

Supplementary Fig. 13 shows our ultrafast transient reflectivity and Kerr rotation measurements in TaAs. All measurements were done in the ultrafast laser with a pulse width ∼40 fs, a central wavelength of 800 nm, and a repetition rate of 80 MHz.

Supplementary Fig. 14 shows the transfer function $h_{\mathrm{prop}}(\Omega)$ and detector response $h_{\mathrm{det}}(\Omega)$.

Using Eq. (9) in the main text and assuming the light absorption nearly the same for different Θ, we can obtain quantitatively $J_{\mathrm{y}}(t)$ and $J_{\mathrm{z}}(t)$, which are shown in Supplementary Fig. **15**.

Supplementary Refrences

[1] Wu, L. *et al.* Giant anisotropic nonlinear optical response in transition metal monopnictide Weyl semimetals, *Nat. Phys.* **13**, 350-355 (2017).

[2] Dadsetani, M. and Ebrahimian, A. Optical distinctions between Weyl semimetal TaAs and Dirac semimetal Na_3Bi: An Ab initio investigation. *J. Electronic Mater.*, **45**, 5867-5876 (2016).

[3] Xu, B. *et al.* Optical signatures of Weyl points in TaAs. *Phys. Rev. B* **93**, 121110 (2016).

[4] Weber, C. P. *et al.* Similar ultrafast dynamics of several dissimilar Dirac and Weyl semimetals. *J. Appl. Phys.* **122**, 223102 (2017)

[5] Kuel, P., Khazan, M.A. and Kroupa, J. Spatiotemporal transformations of ultrashort terahertz pulses. *J. Opt. Soc. Am. B* **16**, 1795-1800 (1999).

[6] Kampfrath, T., Ntzold, J. and Wolf, M. Sampling of broadband terahertz pulses with thick electro-optic crystals. *Appl. Phys. Lett.* **90**, 231113 (2007).
